# LEAFY1 and 2 are required for floral organ development in soybean

**DOI:** 10.1007/s42994-024-00192-2

**Published:** 2024-12-22

**Authors:** Lingshuang Wang, Huan Liu, Lei Chen, Tong Su, Shichen Li, Chao Fang, Sijia Lu, Baohui Liu, Hui Yang, Fanjiang Kong

**Affiliations:** Guangdong Key Laboratory of Plant Adaptation and Molecular Design, Guangzhou Key Laboratory of Crop Gene Editing, Innovative Center of Molecular Genetics and Evolution, School of Life Sciences, Guangzhou University, Guangzhou, 510006 China

**Keywords:** Soybean, *LFY*, Knockout, Floral organ development, Expression analysis

## Abstract

The transition from vegetative to reproductive growth is a vital step for the reproductive success of plants. In *Arabidopsis thaliana*, LEAFY (LFY) plays crucial roles in inflorescence primordium and floral organ development, but little is known about the roles of its homologs in crop plants such as soybean (*Glycine max*). Here, we investigated the expression patterns and functions of the two *LFY* genes (*LFY1* and *LFY2*) in soybean. Both genes were predominantly expressed in unopened flowers and the shoot apical meristem, with *LFY2* having the higher transcript abundance. In an in situ hybridization assay, *LFY* genes produced strong signals in the floral meristem. We next generated *lfy1* and *lfy2* knockout lines. The *lfy2* mutants showed obvious changes in floral organ morphology, but the *lfy1* mutants showed no obvious changes in floral organ morphology or pod development. The *lfy1 lfy2* double mutants displayed more serious defects in floral organ development than *lfy2*, resulting in complete sterility. Gene expression analysis revealed differences in expression of the A-class *APETALA* (*AP*) genes *AP1a* and *AP1b* in the double mutant lines. These results suggest that LFY2 plays an important role in floral organ formation in soybean by regulating the expression of homeotic genes. Our findings increase the understanding of floral development, which could be useful for flower designs during hybrid soybean breeding.

**Supplementary Information:**

The online version contains supplementary material available at 10.1007/s42994-024-00192-2.

## Introduction

The formation of floral organs is crucial for the successful reproduction of angiosperms. In Arabidopsis (*Arabidopsis thaliana*), floral development is largely controlled by the master transcription factor LEAFY (LFY), which plays a key role in promoting floral fate, ultimately resulting in the formation of flowers with four concentric whorls, including sepals, petals, stamens, and carpels (Jin et al. 2020; Meyerowitz et al. 1991). LFY is necessary and sufficient to trigger the onset of flower formation by regulating the expression of floral homeotic genes, such as *APETALA1* (*AP1*), *AP3*, *PISTILLATA* (*PI*), and *AGAMOUS* (*AG*) (Khan et al. 2015; Parcy et al. 1998; Weigel et al. 1992; Winter et al. 2011). Here, LFY directly activates the A-class gene *AP1* throughout the floral primordium, thus triggering floral meristem formation. LFY also activates the B-class gene *AP3* in the second whorl (petals) and the third whorl (stamens) in conjunction with UNUSUAL FLORAL ORGANS (UFO), a component of an SCF (Skp1–Cullin–F-box protein) ubiquitin ligase complex (Chae et al. 2008; Samach et al. 1999). The transcriptional activation of the C-class gene *AG,* in the third whorl (stamens) and the fourth whorl (carpels), depends on the physical interaction between LFY and the pluripotency factor WUSCHEL (WUS) (Laux et al. 1996; Lohmann et al. 2001).

Despite the progress made in understanding the role of LFY in regulating floral development in Arabidopsis, this process remains poorly understood in important crop species, such as soybean (*Glycine max*), a vital legume. Unlike Arabidopsis flowers, a typical soybean flower contains (in order from the outmost to innermost whorl) two bracts, five sepals (the calyx), five petals (one vexil, two wings, and two keels), ten stamens, and a single carpel wrapped by the stamens. The flowers follow a developmental pattern in which more than one whorl is initiated at the same time and alternates with that of the preceding whorl (Tucker 2003). The sepals, petals, stamens, and single carpel are attached at the same level, and the single carpel ultimately differentiates into a gynoecium with an ovary, style, and stigma, which forms a pod-like fruit (Tucker 2003).

Identifying soybean genes involved in regulating floral development, along with their roles in the process, could provide key targets for yield and quality improvement. In recent years, *LFY* homologs have been identified in many species, and these LFY proteins share the same DNA binding specificity and play conserved roles in regulating the development of floral organs (Maizel et al. 2005; Sayou et al. 2014). For example, heterologous expression of the *Medicago truncatula LFY* ortholog *SINGLE LEAFLET1* rescued the floral defects of Arabidopsis *lfy* mutants, and vice versa (Wang et al. 2008). Mutants of the woodland strawberry (*Fragaria vesca*) *FveLFY* ortholog exhibit homeotic conversion of floral organs and reiterative outgrowth of ectopic flowers (Zhang et al. 2023). Rice (*Oryza sativa*) *rfl* mutants (*LFY* ortholog mutants) also display aberrant floral organ identity and the loss of floral meristem determinacy (Kyoko et al. 2015).

Here, we identified two *LFY* homologs in soybean, which we named *LFY1* and *LFY2*. Despite evidence suggesting that *LFY1* and *LFY2* regulate soybean flower development, the two genes have not been genetically characterized. In addition, we generated soybean *lfy* mutants using clustered regularly interspaced short palindromic repeats/CRISPR-associated nuclease 9 (CRISPR/Cas9) gene editing and explored the regulatory functions of *LFY1* and *LFY2* in floral meristem development. Loss of function of *LFY1* or *LFY2* had different effects on floral morphology, and *lfy1 lfy2* double mutants exhibited a complete loss of floral organs, which were replaced by clustered leaf-like structures. Further analysis suggested that LFYs regulate flower development by modulating *AP1a* and *AP1b* gene expression in soybean. These findings enhance our understanding of flower development in this important legume crop.

## Results

### Characterization of *LFY* homologs in soybean

**Fig. 1:**
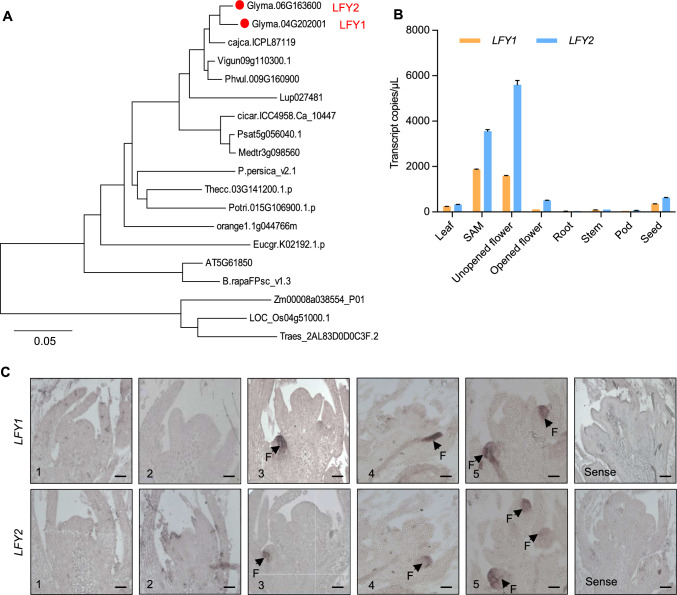
Analysis of *LFY* genes in soybean. **A** Phylogenetic tree of LFY proteins in plants. The two LFY soybean proteins are marked in red. **B** Tissue-specific expression of *LFY1* and *LFY2* in soybean cultivar Williams 82 examined by a droplet digital PCR (ddPCR) assay. Error bars indicate ± SD of three independent replicates. **C** Spatial expression of *LFY1* and *LFY2* in the shoot apical meristems (SAMs) from Wm82 plants at the 1-leaf to 5-leaf stages, as determined by in situ hybridization. Arrows point to the floral meristem. Scale bars = 100 μm

LFY is a plant-specific transcription factor essential for flower development. LFY is one of the few master regulators of flower development, as it integrates environmental and endogenous signals to orchestrate the entire flowering network. We constructed a phylogenetic tree to examine the relationships of LFY homologs in legumes and other plants. These soybean LFYs were positioned much closer to these proteins in legumes, such as *Cajanus cajan*, *Vigna unguiculata*, and *Phaseolus vulgaris*, than to those in other plants (Fig. 1A). As a result, we identified two soybean orthologs named *LFY1* (Glyma.04G202001) and *LFY2* (Glyma.06G163600). LFY1 and LFY2 share high amino acid sequence similarity (94.88%). Both proteins contain a conserved sterile alpha motif domain at their N termini that mediates LFY oligomerization and a conserved Leafy/Floricaula (LFY_FLO) domain at their C termini for DNA binding (Maizel et al. 2005; Sayou et al. 2016), which are characteristics of proteins involved in floral meristem identity (Fig. S1A).

To investigate the soybean expression patterns of *LFY1* and *LFY2*, we performed droplet digital PCR (ddPCR) using root, stem, leaf, shoot apical meristem (SAM), unopened flower, opened flower, pod, and seed tissue to measure the numbers of transcripts produced by the two genes in different organs. Both *LFY* genes were predominantly expressed in unopened flowers and the SAM. *LFY2* had 1.2–4.7 times higher transcript abundance than *LFY1* in all organs examined (Fig. 1B), which is consistent with the results of RT-qPCR (Fig. S1B).

The shoot apical meristem (SAM) is the key organ determining the early formation of the floral meristem, which undergoes cell division and cell differentiation and ultimately develops into flowers (Mayer et al. 1998; Schoof et al. 2000). We therefore examined the localization of *LFY1* and *LFY2* expression patterns by in situ hybridization, using SAMs at the 1-leaf to 5-leaf stages. We designed specific probes targeting the coding sequences of *LFY1* and *LFY2* and performed in situ hybridization assays. Both *LFY1* and *LFY2* continuously produced strong signals using antisense probes in floral meristem from the 3-leaf to 5-leaf stages, suggesting the importance of *LFY* gene expression in the early stages of floral development (Fig. 1C). To further explore the characteristics of soybean LFY proteins, we generated LFY1-GFP and LFY2-GFP (green fluorescent protein) fusion constructs and transiently introduced them into *Nicotiana benthamiana*. Both fusion proteins were localized to the nucleus (Fig. S1C).

### Generation of soybean *lfy* mutants

**Fig. 2:**
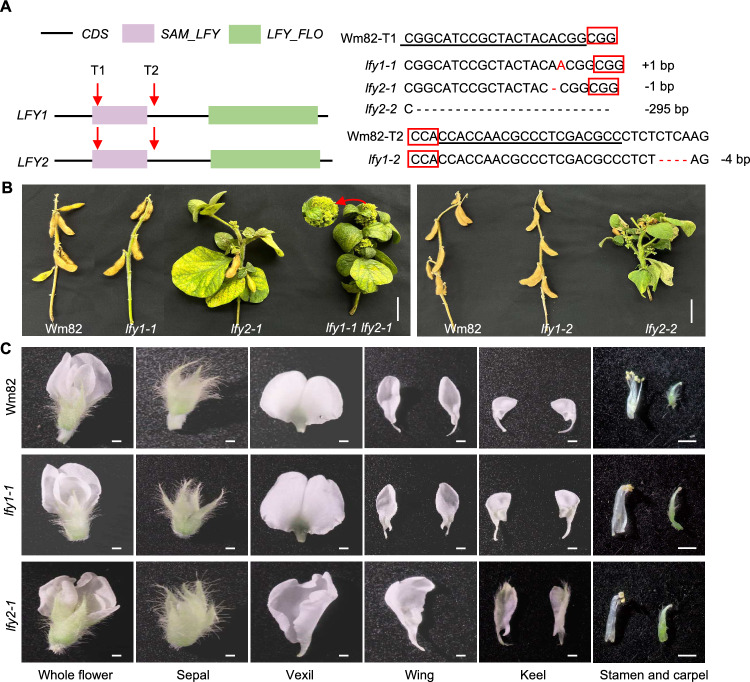
Generation and phenotypes of the soybean *lfy* mutants. **A** Generation of the *lfy1* and *lfy2* mutants by CRISPR-Cas9 gene editing. T1 and T2 represent targets 1 and 2, respectively. Red boxes indicate the protospacer-adjacent motif (PAM) NGG. Red dashes indicate deleted nucleotides. *CDS*, coding sequence; *SAM_LFY*, sterile alpha motif domain; *LFY_FLO*, *LEAFY/FLORICAULA* domain. **B** Whole-plant phenotypes of Williams 82 (Wm82) and the *lfy* mutants. The enlarged image (indicated by a red arrow) highlights the replacement of floral organs by clusters of leaf-like structures in the *lfy1-1 lfy2-1*double mutant plants. Scale bar = 5 cm. **C** Floral organ morphology of Wm82, *lfy1-1*, and *lfy2-1*. Scale bar = 0.1 cm

To elucidate the roles of the soybean *LFY* homologs, we generated knockout mutants of *LFY1* and *LFY2* in the reference cultivar Williams 82 (Wm82) background using CRISPR/Cas9 gene editing. We designed two generic target adaptors to knock out both *LFY*s, simultaneously, with target 1 present in the sterile alpha motif domain and target 2 present at the end of exon 1 (Fig. 2A). We generated stably transformed soybean plants and screened them based on the presence of the *Cas9* gene. Following sequencing analysis, we ultimately obtained two homozygous *lfy1* single mutants (*lfy1-1* and *lfy1-2*), with a 1-bp insertion in target 1 in *lfy1-1* and a 4-bp deletion near target 2 in *lfy1-2*, which both cause frameshifts and translational termination. We also obtained two homozygous *lfy2* single mutants (*lfy2-1* and *lfy2-2*) with a 1-bp deletion in target 1 in *lfy2-1* and a 295-bp deletion in target 1 in *lfy2-2*, both leading to stop codons. These plants no longer contained the Cas9 transgene. The homozygous *lfy1-1 lfy2-1* plants carried the same mutations as *lfy1-1* and *lfy2-1* (Fig. 2A).

### *LFY* genes regulate floral meristem development in soybean

To examine the consequences of the loss of LFY1 and LFY2 function, we grew the *lfy1* single mutants, *lfy2* single mutants, *lfy1 lfy2* double mutants, and wild-type Wm82 in a growth chamber under short-day conditions and observed their phenotypes. Compared to Wm82, *lfy1-1* and *lfy1-2* displayed normal vegetative growth and completely normal morphology of flowers and other organs, and they produced pods with normal fertility (Fig. 2B, C). However, a large proportion of floral meristems in *lfy2-1* and *lfy2-2* failed to produce floral organs, which were replaced by clusters of leaf-like structures on their flanks, leading to severe sterility (Fig. 2B). The successfully developed floral organs exhibited abnormal morphology (Fig. 2C). In Wm82 the flowers included a whorl of five sepals (the calyx) and a whorl of five petals (one vexil, two wings, and two keels). By contrast, *lfy2* showed abnormal floral organ number and shape: *lfy2-1* flowers contained 6–8 sepals with an abnormal vexil, only one wing, and fluffy keel petals (Fig. 2C). The number and morphology of stamens and carpels in successfully developed floral organs did not significantly differ in *lfy1-1* and *lfy2-1* compared to Wm82 (Fig. 2C). We generated *lfy1-1 lfy2-1* double mutants, which failed to produce floral organs and displayed complete sterility, with clusters of leaf-like structures throughout their flanks (Fig. 2B). We therefore generated *lfy1 lfy2* seeds using heterozygous plants.

**Fig. 3:**
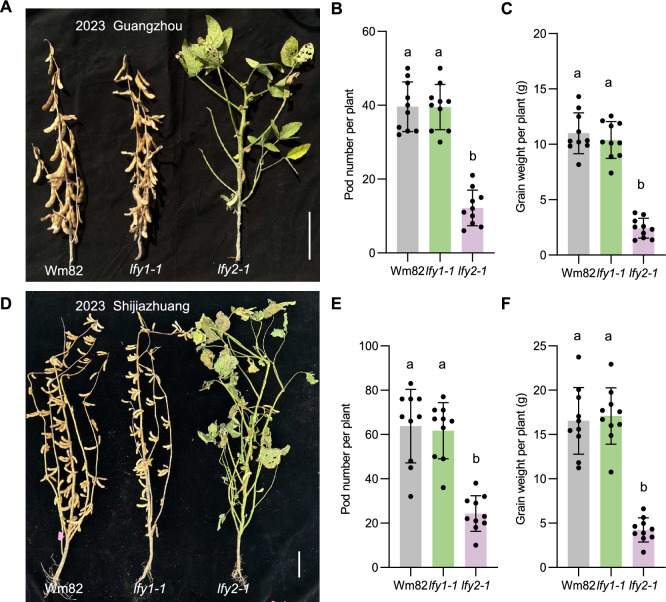
Phenotypes of the soybean *lfy* mutants in the field. **A–C** Phenotypes and productivity of plants grown under natural short-day conditions. **A** Phenotypes of the *lfy1* and *lfy2* single mutants grown the field in a low-latitude region in Guangzhou in 2023. Wm82, Williams 82. Scale bar = 10 cm. **B** Pod number per plant. **C** Grain weight per plant. **D–F** Phenotypes and productivity of plants grown under natural long-day conditions.** D** Phenotypes of the *lfy1* and *lfy2* single mutants grown in a high-latitude region in Shijiazhuang in 2023. Scale bar = 10 cm. **E** Pod number per plant. **F** Grain weight per plant. Ten plants were scored for each phenotype; data are means ± SD, with all individual values shown as dots. Different lowercase letters indicate significant differences between bars (*P* < 0.05, as determined by multiple comparison testing by one-way analysis of variance)

To examine the phenotypes of these mutants under natural conditions, we grew the two single mutants (*lfy1-1* and *lfy2-1*) in the field in natural environments in a low-latitude region in Guangzhou and a high-latitude region in Shijiazhuang, China, and scored the phenotypes at maturity. The *lfy1* and *lfy2* single mutants exhibited phenotypes similar to those in the growth chamber: *lfy1* showed normal plant development, but *lfy2* produced very few mature pods under both natural short-day conditions (Guangzhou) and long-day conditions (Shijiazhuang) (Fig. 3A, D). In the field, *lfy1* did not show altered pod development, whereas *lfy2* produced very few pods and exhibited strongly reduced grain weight owing to the limited number of successfully developed floral organs (Fig. 3). Based on these phenotypes, we proposed a model for soybean in which *LFY1* and *LFY2* regulate floral meristem development, in an asymmetric manner, with *LFY2* playing a more dominant role.

### LFYs regulate *AP1* gene expression in soybean and, thus, control flower development

**Fig. 4:**
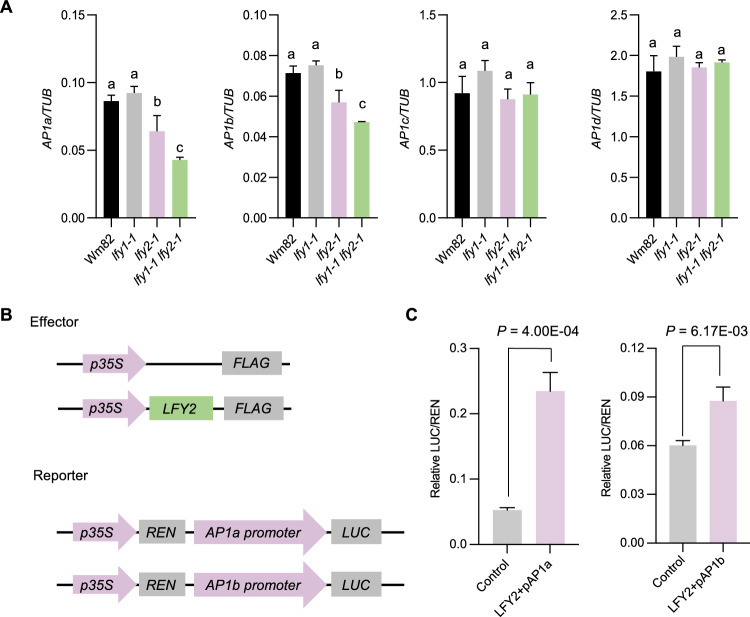
LFYs regulate *AP1a* and *AP1b* expression and thus control flower development. **A** Relative expression levels of *AP1a*, *AP1b*, *AP1c*, and *AP1d* in the SAM of *lfy* mutants isolated at the 4-leaf stage. Wm82, Williams 82. Data shown are relative to the control gene *Tubulin (TUB)*, and error bars indicate ± SD of three independent replicates. Different lowercase letters indicate significant differences between bars (*P* < 0.05, as determined by multiple comparison testing by one-way analysis of variance). **B** Schematic diagrams of the effector construct for *LFY2* and the reporter constructs containing the *AP1a* and *AP1b* promoters. **C** LFY2 promotes *AP1a* and *AP1b* transcription in a dual-luciferase reporter assay in *N. benthamiana* leaves. A Student’s *t*-test was used to generate the *P*-values

LFY directly binds to the *AP1* promoter and regulates its expression in Arabidopsis (Busch et al. 1999; Parcy et al. 1998). *AP1* functions as an A-class gene for floral organ development, as it helps specify carpel, stamen, petal, and sepal identities (Han et al. 2014; Mandel et al. 1992). There are four *AP1* homologs (*AP1a*, *AP1b*, *AP1c*, and *AP1d*) in soybean, and the *gmap1* quadruple mutant exhibits malformed floral morphology (Chen et al. 2020) similar to the morphological changes in *lfy2* flowers. We therefore explored the transcriptional regulatory relationship between LFYs and *AP1* homologs in soybean. First, we performed RT-qPCR analysis to measure the relative transcript levels of *AP1a*, *AP1b*, *AP1c*, and *AP1d* in Wm82 and the *lfy* mutants in their SAMs isolated at the 4-leaf stage. The expression of the four *AP1* homologs showed no significant changes in *lfy1-1* relative to Wm82. By contrast, both *AP1a* and *AP1b* were markedly downregulated in *lfy2-1* and even more strongly downregulated in *lfy1-1 lfy2-1*. However, the expression of *AP1c* and *AP1d* showed no significant changes in *lfy2-1* or even *lfy1-1 lfy2-1* relative to Wm82 (Fig. 4A).

The four soybean *AP1s* belong to two clades: *AP1a* and *AP1b* are grouped closely together, and *AP1c* and *AP1d* share high similarity, pointing to possible functional differentiation between the two homoeologous pairs (Chen et al. 2020). To further verify the regulatory effect of LFY2 on *AP1a* and *AP1b* expression, we performed a transient infiltration assay in *Nicotiana benthamiana* leaves. We expressed the firefly luciferase (LUC) reporter gene under the control of the *AP1a* and *AP1b* promoters and used an LFY2 effector construct (Fig. 4B). LUC activity driven by the *AP1a* and *AP1b* promoters was significantly promoted by LFY2 (Fig. 4C). These results suggested that soybean LFYs affect floral organ development by regulating the expression of *AP1a* and *AP1b*.

## Discussion

### *LFY* orthologs show diverse expression patterns in different species

The SAM, the ultimate source of all aboveground organs, undergoes cell division and cell differentiation that convert it into the inflorescence meristem and then the floral meristem, which ultimately develops into flowers (Hempel and Feldman 1995; Long and Barton 2000; Liu et al. 2009). Spatiotemporal gene expression patterns are an important indicator of gene function, especially for genes involved in development; changes in gene expression patterns are the major drivers of morphological divergence (Carroll 2008; Hoekstra and Coyne 2007; Moyroud et al. 2010). In the model species Arabidopsis, *LFY* was highly expressed in the FM and in all four floral organ primordia, but no expression was detected in the inflorescence meristem or vegetative SAM (Weigel et al. 1992). However, the expression patterns of *LFY* genes are divergent and species-specific. For instance, *FALSIFLORA* (*FA*), the tomato (*Solanum lycopersicum*) ortholog of *LFY*, is highly expressed in the vegetative and sympodial meristem and the floral meristem (Molinero-Rosales et al. 1999). In cucumber (*Cucumis sativus*), *CsLFY* is strongly expressed in the SAM, floral meristem, and floral organ primordia (Zhao et al. 2017). In woodland strawberry, *FveLFYa* is transcribed in the FM, receptacle meristem, carpels, and SAM (Zhang et al. 2023). Here, we established that, in soybean, *LFY1* and *LFY2* were highly expressed in unopened flower and the SAM, as revealed by ddPCR analysis (Fig. 1B). We further localized *LFY1* and *LFY2* expression to the FM by in situ hybridization (Fig. 1C), indicating their roles in maintaining the determinate initiation of flowers in soybean.

### *LFY1* and *LFY2* play asymmetric roles in soybean

Based on phenotypes, the *lfy1* and *lfy2* mutations had different effects on floral morphology. Loss of function of *LFY1* appeared to have no effect on flower development. However, the *lfy1 lfy2* double mutant exhibited stronger floral organ defects than the *lfy2* single mutants (Fig. 2B), suggesting that *LFY1* plays a less important role in this process. The differential expression levels of genes and protein sequences are closely correlated with the functional divergence of duplicated genes (Ganko et al. 2007). LFY1 and LFY2 share extremely similar amino acid sequences, especially the sequences of their two important N-terminal and C-terminal domains, which are completely identical (Fig. S1A), pointing to a possibly conserved function of the two proteins. However, ddPCR analysis showed that soybean plants contain more copies of the *LFY2* transcript, with 1.2–4.7 times higher transcript levels than *LFY1* in all organs examined, which is consistent with the results of RT-qPCR (Figs. 1B and S1B). Based on these findings, we suggest that the differential functions of soybean *LFY1* and *LFY2* might be associated with their different transcript levels, which could be related to either upstream regulatory proteins or *cis*-regulatory DNA elements (Carroll 2008; Hoekstra and Coyne 2007). This phenomenon is worth exploring in the future.

In summary, our study provides insight into the *LFY* homologs in soybean. The complete loss of function of soybean *LFY*s in the double mutant led to the disappearance of floral organs, which were replaced by clusters of leaf-like structures on the flanks of the plant, as well as complete sterility. The functions of *LFY* genes are generally conserved among species. Soybean LFYs affect floral organ development by regulating the expression of *AP1a* and *AP1b*. These findings provide a basis for further investigating the LFY-mediated regulation of floral organ development in soybean.

## Materials and methods

### Plant materials and growth conditions

The soybean cultivar Williams 82 (Wm82) was used for gene expression analysis and for stable genetic transformation. Plants were cultivated under short-day conditions in an incubator with a 12-h light/12-h dark cycle, relative humidity of 70% ± 10%, and light intensity of 500 µmol m^−2^ s^−1^. The mutants used for phenotyping were grown in the field under natural conditions in a low-latitude region in Guangzhou (23°8′N) and a high-latitude region in Shijiazhuang (37°27′N).

### Phylogenetic analysis and sequence alignment

The amino acid sequences of LFY1 and LFY2 in Wm82 were obtained from Phytozome 13 (https://phytozome-next.jgi.doe.gov/) and aligned using DNAMAN software to create multiple sequence alignments. The AtLFY (*AT5G61850*) protein sequence from Arabidopsis was retrieved from TAIR (https://www.arabidopsis.org/index.jsp). The LFY-like protein sequences from *Medicago truncatula* (*Medtr3g098560*), *Cicer arietinum* (*cicar.ICC4958.Ca_10447*), *Phaseolus vulgaris* (*Phvul.009G160900*), *Vigna unguiculata* (*Vigun09g110300.1*), *Cajanus cajan* (*cajca.ICPL87119*), *Lupinus angustifolius* (*Lup027481*), and *Pisum sativum* (*Psat5g056040.1*) were obtained from the Legume Information System (https://legumeinfo.org/). The protein sequences from *Prunus persica* (*P. persica v2.1*), *Theobroma cacao* (*Thecc.03G141200.1.p*), *Populus trichocarpa* (*Potri.015G106900.1.p*), *Citrus sinensis* (*orange1.1g044766m*), *Eucalyptus grandis* (*Eucgr.K02192.1.p*), *Brassica rapa* (*B. rapaFPsc v1.3*), *Zea mays* (*Zm00008a038554_P01*), *Oryza sativa* (*LOC_Os04g51000.1*), and *Triticum aestivum* (*Traes_2AL83D0D0C3F.2*) were obtained from Phytozome 13. Phylogenetic analysis was performed using MEGA11 with the neighbor-joining method and with a bootstrap test comprising 1000 replicates.

### Vector construction and genetic transformation of soybean

Two target sequences were designed to knock out *LFY1* and *LFY2* simultaneously. The target sequences were subcloned into single-guide RNA (sgRNA) expression cassettes and integrated into the pYLCRISPR/Cas9-DB vector as previously described (Ma et al. 2015). The resulting CRISPR/Cas9 plasmid was introduced into *Agrobacterium tumefaciens* strain EHA101 for stable soybean transformation using the cotyledonary node method as described previously (Flores et al. 2008). To identify transgene-positive mutants, DNA was extracted from the leaves of plants, and PCR was performed using specific primers to detect *Cas9* and primers to detect the target sequences of *LFY1* and *LFY2*. All of these primers are listed in Table S1.

### RNA extraction and RT-qPCR

Different organs of Wm82 and the SAMs of mutants were separately collected, and total RNA was extracted from the samples using a Nuclei Acid Extraction kit following the manufacturer’s instructions (CoWin Biotech). cDNA was synthetized from the RNA using a First‐strand cDNA Synthesis kit (Takara). Quantitative reverse-transcription PCR (RT‐qPCR) was performed using a Real‐time PCR kit (Takara) in a Roche Light Cycler 480 instrument (Roche Molecular Biochemicals). The expression levels of the target genes were normalized to those of *β-Tubulin*. Each experiment was performed using three biological replicates from RNA samples extracted from three independent plants, each with three technical replicates. All primers used for RT‐qPCR are listed in Table S1.

### Droplet digital PCR

The absolute expression levels of *LFY1* and *LFY2* in different organs of Wm82 were analyzed by droplet digital PCR (ddPCR). ddPCR was performed in a 20-ml volume containing 10 mM of each target-gene primer and probe, 5 ml ddPCR Supermix (dUTP), 10 ng cDNA, and distilled water. Nanoliter-sized droplets were generated and PCR was performed in a MicroDrop-100A instrument (Forevergen, Guangzhou, China) according to the manufacturer’s instructions. After PCR amplification, the reactions were placed in a MicroDrop-100B instrument (Forevergen), and the droplets were analyzed with QuantDrop software. Each sample was obtained from three individuals, and the data were analyzed with three technical replicates. The primers and probes are listed in Table S1.

### In situ hybridization assay

SAMs were collected from plants at the 1-leaf stage to 5-leaf stage, and sample preparation and mRNA in situ hybridization were performed as described by De Block and Debrouwer (1993) and Liu et al (2022). To generate RNA probes for *LFY1* and *LFY2*, specific fragments were amplified with the primers listed in Table S1 and integrated separately into the pSPT18 vector under the control of the T7 or SP6 promoter with RNA polymerase using a DIG RNA Labeling kit (Roche). Digoxigenin-labeled sense and antisense probes were obtained from linear pSPT18 vectors according to the manufacturer’s protocol. Hybridization signals were detected and photographed under a fluorescence microscope (Zeiss, Axio Imager A2).

### Subcellular localization

The coding sequences of *LFY1* and *LFY2* from Wm82 were amplified using the primers listed in Table S1, and the fragments were cloned into pTF101‐GFP under the control of the cauliflower mosaic virus (CaMV35S) promoter. The constructs were transformed into *Agrobacterium tumefaciens* strain GV3101 and infiltrated into 4-week-old *Nicotiana benthamiana* leaves. The leaves were incubated for 48–72 h and observed and imaged using a LSM 800 laser scanning confocal microscope (Zeiss, Oberkochen, Germany).

### Transient infiltration assay

The experiment is done according to Pei et al (2023). The ~ 3 kb promoter sequences of *AP1a* and *AP1b* were amplified from Wm82 and introduced into the pGreen0800‐LUC/REN vector (the gene encoding *Renilla* luciferase [REN] under the control of the 35S promoter was used as the internal control) to generate the 35S:REN‐pAP1a:LUC and 35S:REN‐pAP1b:LUC reporter constructs. The coding sequence of *LFY2* was introduced into the pTF101-FLAG vector to generate the 35S:LFY2‐FLAG effector construct. The constructs were introduced into *Agrobacterium tumefaciens* strain GV3101, and equal volumes of cell suspensions were co‐infiltrated into fresh *N. benthamiana* leaves. The empty vector mixed with effector constructs were used as controls. The leaves were incubated for 48–72 h. At least three leaves from independent *N. benthamiana* plants were infiltrated. LUC and REN activities were measured using a Luciferase 1000 Assay System (Promega) and a Renilla Luciferase Assay System (Promega), respectively. Transcriptional activity was expressed as the ratio between LUC and REN activity. The primers used in this assay are listed in Table S1.

## Supplementary Information

Below is the link to the electronic supplementary material.

Supplementary file1 (PDF 182 KB)

## Data Availability

All data supporting the findings of this study are available within the article and supplementary files.
