# Supplementary material for: LEAFY1 and 2 are required for floral organ development in soybean
Source: aBIOTECH. 2024 Dec 22;6(1):12–21. doi: 10.1007/s42994-024-00192-2 (PMC11889283; doi:10.1007/s42994-024-00192-2)
Supplement: Supplementary file 1 — Supplementary file1 (PDF 182 KB) [file 42994_2024_192_MOESM1_ESM.pdf]

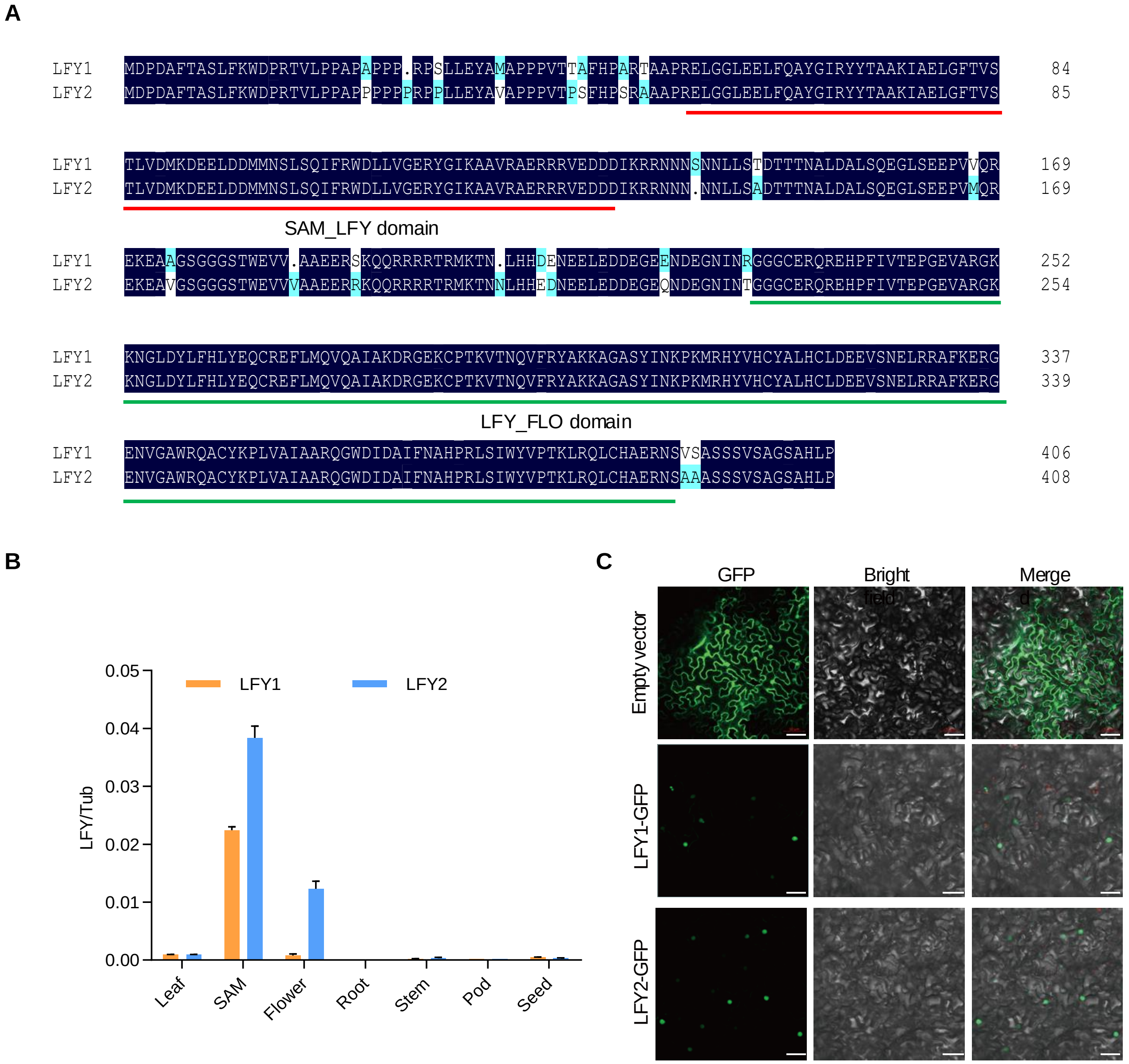

Fig. S1 Analysis of soybean *LFY* genes by amino acid sequence alignments and tissue-specific expression

An Amino acid sequence alignments of LFY1 and LFY2 of soybean. The red underline represents SAM_LFY domain and the green underline represents LFY_FLO domain. B Tissue-specific expression of *LFY1* and *LFY2* in soybean cultivar Williams 82 generated by the RT-qPCR. Data shown are relative to the control gene *Tubulin*, and error bars indicate ± SD of three independent replicates. C Subcellular location of LFY1-GFP and LFY2-GFP in Nicotiana tabacum. Scale bars = 50 μm.
